# Abstract of the Proceedings of the Buffalo Medical Association

**Published:** 1871-01

**Authors:** Wm. C. Phelps

**Affiliations:** Secretary; Buffalo


### ART. III.—*Abstract of the Proceedings of the Buffalo Medical Association.*

BUFFALO, December 6th, 1870.

The meeting was called to order by the Vice-President.

Reading of minutes of last meeting dispensed with.

Dr. Gay wished to call the attention of the Association to a subject which, to his knowledge, had never yet been introduced into our discusssion, viz: hour-glass contractions. It had been asserted by some authors that those contractions do not exist, and by others that they do occur, but much less frequently than is supposed; that the contraction of the os uteri, around or over the hand or wrist, is often mistaken for contraction of the circular fibers of the muscles of the body of the uterus, and the mistake therefore made of calling the case one of hour-glass contraction; when, in fact, the contraction is not of this kind or species at all. But there can be no doubt at all of the occasional existence of these contractions, in which a case retained placenta is always presupposed and and admitted. There can be no doubt of the existence of what is called hour-glass contraction before the expulsion of the placenta. Can this abnormal condition of the uterus be present before either the placenta or child is expelled? To this question the doctor would like to call attention of members of the Association. He had examined several authorities, but could obtain no light upon the subject matter; yet, from personal experience and observation, he is convinced that hour-glass contraction may, and does, exist prior to the expulsion of the child, and related a case in point. He would state simply the facts and leave the case with the evidence adduced of the opinion expressed, that hour-glass contraction may exist before the expulsion of the child, for the consideration of members present, and for them either to accept or reject the evidence. He had visited a patient on November 2d, who had been in labor about forty-eight hours. She had had five or six difficult labors, giving birth to still-born children. She had been attended in her present labor, since five o'clock in the morning, by Drs. Diehl and Wetmore. Up to that hour she had been attended by a professional midwife.

The vertex presented; forceps had been used but would not retain their hold upon the head. Craniotomy was then performed, and Drs. Diehl and Wetmore becoming fatigued, and somewhat exhausted, sent, at nine a. m., for Dr. Gay, who, after failure with the forceps, introduced his left hand—the patient being under the full influence of chloroform—to turn the child. Seizing the foot of the child he found he could not bring down the foot, nor withdraw his hand, on account of the constriction of the circular fibers of the muscles of the uterus. He then introduced the blunt hook with which the leg was drawn down; any effort to remove the hand from the uterus cavity would excite so strong muscular contractions that it became impossible while the hand was closed—by opening the hand he was able to remove it. The constricted portion of uterus appeared to be the size of a rope one inch in diameter, felt as hard as bone, and at first was mistaken for bone; but observing its contraction and dilatation, the character of the tissue was determined. Its contractile power was so forcible, that the pain inflicted upon the wrist, at each uterine contraction, was almost insupportable.

While the hand was in this trap, he declared to his associates that he had hour-glass contraction, and took time to study the relative position of the uterus and its contents. He was able to do this, there being no uterine contractions only when he attempted to withdraw the hand from the maternal organs. He found that the head of the child was below the rope-like and constricted muscles, that there was an upper and lower chamber, and that the uterine contraction was not that of the os uteri but existed above it. In this he could not be mistaken.

The woman was delivered of a still-born child. The anæsthetic effects of the chloroform were followed and maintained by ether.

This woman unquestionably had a mal-formed pelvis; the promontory of the sacrum projected forwards so as to reduce the conjugate diameter to probably three inches. The doctor also stated that, after the leg had been brought down, the child could not be turned by the strongest traction. The method adopted was to introduce his right hand against the head of the child crowding it up through the hour-glass contraction while Dr. Wetmore made traction with the leg. The female made a good recovery.

Dr. Rochester thinks that the hour-glass contraction is caused by injudicious traction on the cord in attempts to remove the placenta. His practice is to wait for a contraction of the uterus, which may be hastened by grasping with the hand the fundus of the uterus, and then remove the placenta. By this course post-partum hemorrhage is avoided, which often occurs when the placenta is removed immediately after delivery, and before the uterus has recovered its contractile power. An experience in near three thousand accouchments has proved this to be the best course to pursue.

Dr. Miner remarked that, in the earlier years of his practice, he often met what he believed to be hour glass contraction of the uterus, but for the last fourteen years had not been troubled with it. His course, after delivery, is to remove the placenta, and he has not, as a consequence, observed post-partum hemorrhage. Can see no advantage in waiting longer than the usual interim of pain, when by making careful traction upon the cord, contraction is obtained, and placenta easily and safely removed. If this does not succeed, he would introduce hand and remove placenta in manner described by Prof. Rochester.

Dr. Cronyn was glad that this subject had been brought before the Association. When he first began the practice of medicine, following the teaching of his professor, he used to wait, but since the third year of practice he has followed the opposite course, and removes at once the placenta. After the child is removed, the placenta becomes a foreign body in the uterus, and is often found grasped by the neck of the womb, particularly if ergot has been given. Hemorrhage may or may not occur when this is the case, the contraction of the uterus being irregular. Was called to a case in the city in which the after-birth was retained, and advised non-interference. Ergot had been given, which contracts the lower part of the uterus as well as the fundus, and chloroform does not relieve the contraction. In a recent discussion before the Obstetrical Society of London, it was shown that in cases of retained placenta, before the fifth month, it should be let alone.

Dr. Rochester said that he never leaves a patient till the afterbirth is delivered, which he does by seizing it, and, by a twisting motion, detatching and removing it. Pulling on the cord should be avoided, as it is liable to cause inversion of the uterus. When hemorrhage follows detachment of the placenta, it is indicated by paleness and a peculiar constant pain, which is felt all around the uterus. In this case the hand should be introduced and the clot removed. Dr. Rochester was called to attend a severe case of diphtheria, occurring in a woman in the eighth month of pregnancy. About a week after recovery she miscarried. When called to see her, found the os somewhat dilated and about a foot of the cord protruding. All the usual means of replacing it were used, but did not succeed. The labor went on slowly, and when dilatation was sufficient found a face presentation, the chin being under the pubes. The forceps, after much difficulty, because of the cord, were applied, but delivery could not be effected. Craniotomy was then performed, the child delivered, and the mother recovered.

Dr. Gay related a case of difficult labor, the report of which is suggested by the case just reported by Dr. Rochester.

Mrs. B., aged twenty-six years, primipara, had severe labor pains at the expiration of the usual period of nine months utero-gestation, lasting an hour or more, then continuing only at intervals, and finally stopping absolutely. Near the completion of ten months utero-gestation, pains came on again, some of which were severe. On examination the os was found undilated: her pains for forty-eight hours thereafter were capricious, sometimes becoming severe and again going off and not returning for some hours. The os was now dilatable, two fingers could easily be introduced through it. The doctor thought it time the woman was delivered, and resolved to deliver her—he accordingly applied forceps, while yet the os was hardly open enough to admit them, from time to time making traction and some progress, although there was no indication of labor pains. After three hours exertion he delivered this woman of a still-born child, its face presenting. Craniotomy was not thought advisable, while some degree of progress was observable at each effort made with the forceps, and so long as any hope existed of saving the life of the child.

The head measured, in circumference, fifteen and three-fourth inches, length of child two feet, minus one-fourth inch; weight, thirteen and one half pounds.

Dr. Gay was hardly willing to concede that his patient had gone over her time more than two weeks; but the patient and her intimate friends could not be dissuaded from their belief that she had gone over her time fully one month, and this undoubtedly was the fact. There were no normal labor pains during the delivery.

Dr. Rochester reported the following case of severe idiopathic tetanus cured by hydrate of chloral. In June last was called to visit a boy about nine years of age, who had suddenly been seized by a tetanic spasm. The jaws were contracted to one-eighth of an inch, sardonic expression, eyelids closed. He had got wet; next morning complained of a stiff neck. On the second he could swallow only with great difficulty, and the next day was worse. July 10th, had a convulsion; pulse 100 and regular, but respiration was imperfect. There was no tenderness of the spine, nor any disorders of digestion. A warm bath was ordered, and an enema of turpentine and croton oil, alternated with the bath, until the bowels were thoroughly evacuated. Morphine, gr. 1-4; and atropia, gr. 1-32; were then given hypodermically. Saw the patient again in a few hours, and found no convulsion, but the jaws were still set—repeated the injection of atropia, gr. 1-32. The second day there was no convulsion. I then crowded the atropia, giving five doses of gr. 1-32. Chapman's ice bag was applied to the spine, and was very grateful to the patient. The third day brought no change in his condition, and hydrate of chloral was resorted to; as he could not swallow, it was given by enema, grs. 10, in one ounce of mucilage. Sleep was produced.

Continued the chloral, alternating it with beef essence. The fourth day another fit occurred, during which the tongue was bitten. This recurred for two days following, then there was no recurrence till the fourth day. The chloral was continued during this time, and till July 1st, six hundred grains having been given in all. The administration of this large amount caused no unpleasant symptoms, but the spasms were controlled. The chloral was continued till August 4th, two hundred grains being given during this time, making in all, from July 13th till August 4th, eight hundred grains. This large amount was retained and absorbed, and a cure was effected. From July 13th till August 1st the ice, wrapped round with a cloth, was continually applied, and was so grateful to the patient that, if removed for a short time, he would beg to have it replaced ; of itself, however, it was not sufficient to control the convulsions.

Dr. Miner remarked that, according to his experience, most cases of acute tetanus die, and spoke of the difficulty of determining the influence of medicines in cases which recover. It had been long noticed that patients suffering from tetanus, who, from any cause, did not soon die, the cases becoming in a certain sense chronic, often recover. He spoke of a case, which was reported at the time, in which a patient remained rigid for three months, and recovered. Dr. Rochester's case was very instructive, and the result indicated the great value of chloral.

Dr. Gay remarked that the case was interesting and the termination pleasant. He regarded the ice box as a valuable agent in such cases. Thinks chloral will never come into general use, because of its large dose and unpleasant taste.

Dr. Cronyn remarked upon the value of chloral, that it should immortalize the name of its discoverer, as chloroform had immortalized the name of Simpson. Had a chronic case of spasm, which he was in the habit of anticipating by the hypodermic injection of morphia. Chloral, in the dose of thirty grains, was given ; and, in addition, ten grains, three times daily, were ordered ; recovery soon followed this course of treatment.

Dr. Miner moved that Dr. S. C. Bateman be invited to attend the meetings of the Association during his stay in the city. Adopted.

Dr. Miner remarked that a large number of cases of diphtheria or diptheritic croup had occurred in certain portions of the city, which were very fatal. A case seen with Dr. Jansen that evening was then dying. Saw a second case with Dr. Mixer, the patient being a girl five or six years of age, who had been feeble for some time At the earnest solicitation of her friends, and after the chances were fully explained, the operation of tracheotomy was performed, and the tracheal tube was introduced. For a time she rallied, and awakened in the minds of friends hopes of recovery. She recognized them, breathed easy, and was very comfortable during the next day. The second day the tube became filled with the false membrane, and the child died forty-eight hours after the operation He had seen five similar cases, in connection with other physicians, during the month, and thought it must be in unusual frequency. So far as treatment is concerned, he would say that he had used, from time to time, all the various remedial agents which were said to be useful. Had given tonics, stimulants and alteratives; had used the local applications made and recommended by physicians, from nitrate of silver down to the spray of lime water, and had repeatedly introduced the tracheal tube. In reviewing his cases, both those he had treated himself, and those he had seen with other physicians, he could not say that any method of treatment yet proposed was of any avail whatever, in the treatment of diphtheretic exudation into the trachea.

He was not *sure* that any treatment he had ever instituted was, in a single case, of any benefit. Diphtheria, when confined to fauces, was for the most part without danger, as it had appeared under his observation ; and, when invading the trachea, it was almost uniformly fatal. Tracheotomy was successful when made in catarrhal, but not when made in true diphtheritic croup, good observers to the contrary notwithstanding ; while, as to medicine, his own observation made him believe that, in all forms thus far proposed, it was entirely useless.

Dr. Gay had been informed by a friend that he had lost four cases of this formidable disease. In his own practice had a patient with tonsilitis complicated with diphtheria ; the neighboring glands were swollen, but the patient was not very sick. Under suitable treatment got better and sat up in bed, but died that night suddenly. A brother of this patient had a wound which would not heal. Like the other was not much sick, and no evidence of diphtheria was present, but he died suddenly like the other.

Adjourned.

WM. C. PHELPS, *Secretary.*

